# Supplementary material for: Components from the Human c-myb Transcriptional Regulation System Reactivate Epigenetically Repressed Transgenes
Source: Int J Mol Sci. 2020 Jan 14;21(2):530. doi: 10.3390/ijms21020530 (PMC7014047; doi:10.3390/ijms21020530)
Supplement: Supplementary file 1 [file ijms-21-00530-s001.zip › ijms-653085 sup/Sub5 - 2019MYB - Supplemental (final v2).pdf]

Components from the human c-myb transcriptional regulation system reactivate epigenetically repressed transgenes

Cassandra M. Barrett, Reilly McCracken, Jacob Elmer, and Karmella A. Haynes

Supplementary Information

Table S1 is provided as a separate Microsoft Excel file

Table S2. Activation-associated protein (AAP) accession numbers and primers
Table S3. dCas9-MYB sgRNA targeting sequences
Table S4. Publicly accessible plasmids used in this study
Table S5. Primers for qPCR to quantify Gal4-AAP mRNA

Figure S1. Histone modifications associated with activation-associated proteins (AAPs) used in this study
Figure S2. Design and construction of activation-associated protein (AAP) -Gal4 fusions
Figure S3. Measurement of luciferase reporter expression within open chromatin after exposure to Gal4-AAP fusions
Figure S4. Additional trial of time course experiments with Gal4-AAP-expressing cells
Figure S5. Annotated motifs within the MYB protein
Figure S6. MTT cell viability assays
Figure S7. Targeting dCas9-MYB to a repressed CMV-GFP transgene in HEK 293 cells

Table S2. Activation-associated protein (AAP) Accession Numbers and Primers. Capitalized nucleotides indicate overhangs for the addition of XbaI and NotI sites for cloning.

AAP	Ref.	UniProt ID	Isoform	NCBI RefSeq	Amplification primers (5' .. 3')
VP64 (4xVP16)	[S1]	n.a.	-	-	F: CCTTATCTAGAgacgctttggacgacttcga<br>R: CTTAGCGGCCGacaacatgtccaagtcgaagt
(NKκB)-p65	[S2]	Q04206	-	NM_001145138.1	F: CCTTATCTAGAtacctgccagatacagacga<br>R: CCTTAGCGGCCGatctcagccctgctgagtcag
MYB	[S3]	P10242	Isoform 1 P10242-1	NM_005375.2	F: GGCCTTATCTAGccagctgccgcagccattca<br>R: AATTAGCGGCCGcccacccggggtagctgcat
ATF2	[S4]	P15336	Isoform 1 P15336-1	NM_001256090.1	F: CCTTATCTAGAgagatgacactgaaatttgg<br>R: CCTTAGCGGCCGaggatcttcgttagctgctc
p300	[S4]	Q09472	Q09472-1	NM_001429.3	gBlock from IDT
KAT2B (PCAF)	[S5]	Q92831	Q92831-1	NM_003884.4	F: CCTTATCTAGActcaaccagaaaccaaacaa<br>R: CCTTAGCGGCCGatttagctcacatcccatta

KMT2A (MLL1)	[S6]	Q03164	Isoform 1 Q03164-1	NM_005933.3	gBlock from IDT
KMT2C (MML3)	[S6]	Q8NEZ4	Isoform 1 Q8NEZ4-1	NM_170606.2	gBlock from IDT
KMT2D (MLL2, 4)	[S6]	O14686	Isoform 1 O14686-1	NM_003482.3	gBlock from IDT
KMT2E (MLL5)	[S6]	Q8IZD2	Isoform 1 Q8IZD2-1	NM_182931.2 (variant 1)	F: GCGCTCTAGAaatttggataaagagagggc<br>R: AATGCGGCCGcttcattactaataggagtt
SETD1A (SET1)	[S6]	O15047	O15047-1	NM_014712.1	F: CCTTATCTAGAaagaagctccgatttggccg<br>R: CCTTAGCGGCCGagtttagggagccccggcagc
SETD1B (SET1B)	[S6]	Q9UPS6	Isoform 1 Q9UPS6-1	No RefSeq number	gBlock from IDT
SETD7 (SET7/9)	[S7]	Q8WTS6	Q8WTS6-1	NM_030648.2	gBlock from IDT
PRMT5	[S8]	O14744	Isoform 1 O14744-1	NM_006109.4	F: ATGCTCTAGAatggcggcgatggcggtcgg<br>R: ATGCGCGGCCGcgaggccaatggtatatgagc
FOXA1	[S9, S10]	P55317	P55317-1	NM_004496	F: GGCTCTAGAatgttaggaactgtgaagatgga<br>R: ATTGCGGCCGccaaggaagtgtttaggacgg
SMARCA4 (BRG1)	[S11]	P51532	Isoform 1 P51532-1	NM_001128844.1 (variant 2)	F: CCTTATCTAGAatgtccactccagacccacc<br>R: CCTTAGCGGCCGgctgctgtccttgtacttga

Table S3. dCas9-MYB sgRNA targeting sequences. The targeting sequences for g46, g31, g32, and g25 have been used in our previous work [S12].

**Transgene**	**Cell line**	**Target Name**	**Targeting Sequence**
Tk-Luciferase	HEK 293 Gal4-EED/luc [S13]	g46	5' cctgcataagcttgccacca 3'
		g31	5' cgaggtgaacatcacgtacg 3'
		g32	5' cctctagaggatggaaccgc 3'
		g25	5' accgtagtgtttgtttccaa 3'
CMV-GFP	HEK 293 GFP insert site 3-3 (Chang Liu, UC Irvine, unpublished)	L1	5' ctctgtcacaggactcagcc 3'
		L2	5' gggcggtaggcgtgtacggt 3'
		L3	5' cactggtgtcgtccctattc 3'
		L4	5' cttcttcaagtccgccatgc 3'

Table S4. Publicly accessible plasmids. Annotated sequences can be viewed online in the Haynes lab Benchling collection "DNA-Binding Fusion Transcriptional Regulators" at https://benchling.com/hayneslab/f_/5wovkOaK-gal4-dna-binding-fusion-transcription-regulators/?sort=name.

**Name**	**DNASU Accession #**
MV14	-
MV14_VP64	HaCD00812388
MV14_p65	HsCD00812387
MV14_MYB	-
MV14_ATF2	HsCD00833013
MV14_p300	HsCD00833014
MV14_KAT2B	HsCD00833015
MV14_KMT2A	HsCD00833016
MV14_KMT2C	HsCD00833018
MV14_KMT2D	HsCD00833017
MV14_KMT2E	-
MV14_SETD1A	HsCD00833019
MV14_SETD1B	HsCD00833020
MV14_SETD7	HsCD00833021
MV14_PRMT5	-
MV14_FOXA1	-
MV14_SMARCA4	HsCD00833022
pX330g_dCas9_MYB	-

Table S5. Primers for RT-qPCR

**Target**	**Amplification primers (5' .. 3')**
mCherry (Gal4-AAP transcripts)	F: gctccaaggcctacgtgaag<br>R: aagttcatcacgcgctccca
TBP (housekeeping gene)	F: caggggttcagtgaggtcg<br>R: ccctgggtcactgcaaagat

	AAP	Known Interactors								Expected Modifications in PRC Chromatin					
		HATs	HMTs	Coactivators	Kinases	Ubiquitinases	Structural	HDACs	DNMTs	H3K27ac	H3S28p	H3 / H4 Kac	H3K4me	H3R17me	H3R26me
Transcriptional activation	(NFκB)-p65	3	2	5	2			2		PE		A	A		
Transcriptional activation	VP64 (4xVP16)	1		4								A			
Transcriptional activation	MYB	2		5	1	1				PE		A			
Histone acetylation	ATF2	3		3	1					PE	PE	A			
Histone acetylation	P300	2	2	9						PE		A	A	A	A
Histone acetylation	KAT2B (PCAF)	8		4				1		PE		A	A		
H3 MT	KMT2A (MLL1)	2		5								A	A		
H3 MT	KMT2D (MLL2)	1	9	2								A	A		
H3 MT	KMT2C (MLL3)	1	7	1			1					A	A		
H3 MT	KMT2E (MLL5)	1	8		2		1						A		
H3 MT	SETD1A (SET1)		10			1	2						A		
H3 MT	SETD1B (SET1B)		10		1		1						A		
H3 MT	SETD7 (SET7/9)		2	4			2		1				A		
H4 MT	PRMT5 (ANM5)		3	2			2		1			A			
CR	SMARCA4 (BRG1)	1		12				1		PE					
PF	FOXA1 (HNF-3A)			3				1							

Figure S1. Histone modifications associated with activation-associated proteins (AAPs) used in this study. Interaction partners determined by STRING analysis are listed in Supplementary Table S1. Previous work that characterized each AAP is cited in Supplementary Table S2. H3 MT = histone H3 methyltransferase, H4 MT = histone H4 methyltransferase, CR = chromatin remodeler, PF = pioneer factor, PE = Polycomb eviction, A = transcriptional activation.

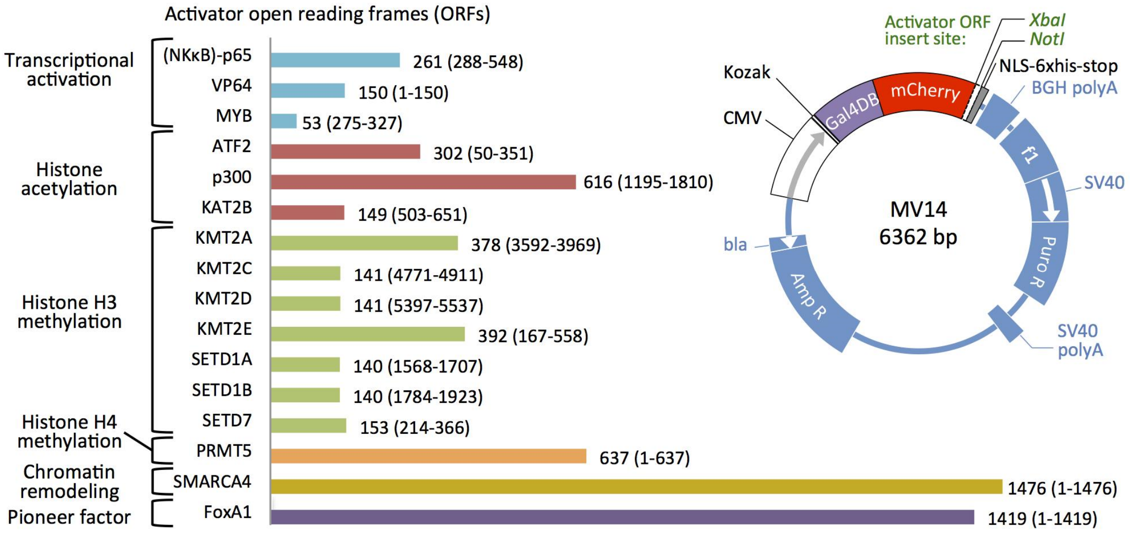

Figure S2. Design and construction of Activation-associated protein (AAP) -Gal4 fusions. Amino acid lengths are indicated, as well as domain location within the full length wild-type sequence (Supplementary Table S2). ORFs were cloned into MV14 to express a Gal4-AAP fusion protein from a cytomegalovirus (CMV) promoter. Gal4-AAPs are expressed with a C-terminal nuclear localization signal (NLS) and a 6X histidine tag. MV14 expresses puromycin resistance to enable selection of Gal4-AAP positive cells.

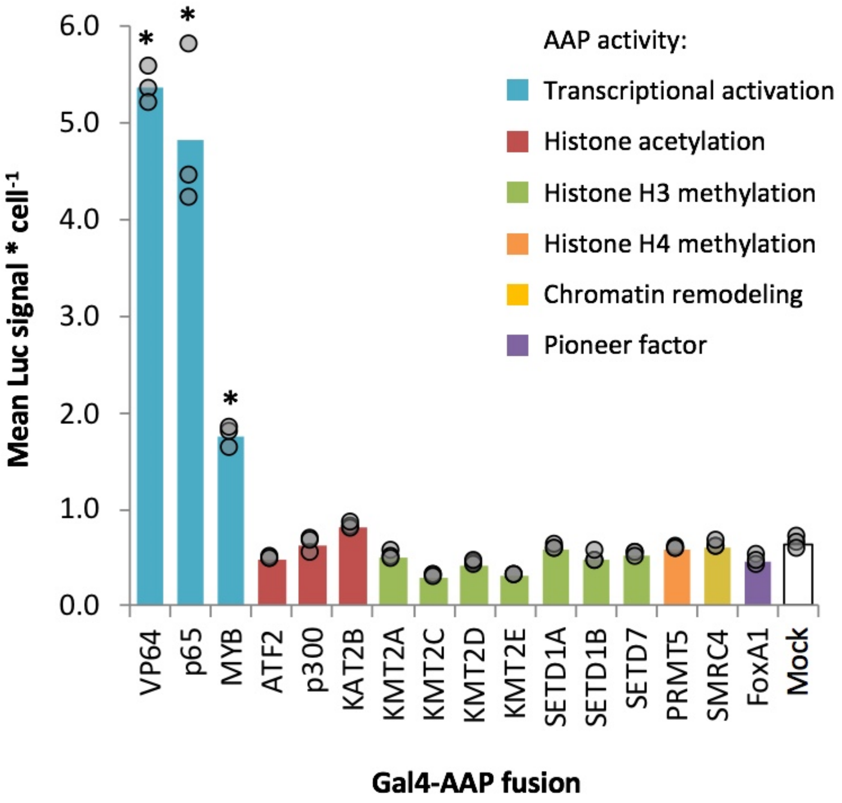

Figure S3. Measurement of luciferase reporter expression within open chromatin after exposure to Gal4-AAP fusions. Luc14 cells carry the same *Tk-luciferase* locus at the same chromosome site as Gal4-EED/luc cells, but without Gal4-EED. Therefore, the site is PRC-depleted and basal levels of expression from *Tk-luciferase* are higher than in Gal4-EED/luc cells (Figure 2). Luc14 cells were transfected with each Gal4-AAP fusion plasmid. Three days after transfection luciferase signal was measured. Each circle in the bar graph shows the mean luciferase (Luc) signal for a single transfection, divided by cell density (total DNA, Hoechst staining signal). Bars show means of three transfections. Asterisks (*) = $p < 0.05$ compared to mock-transfected cells.

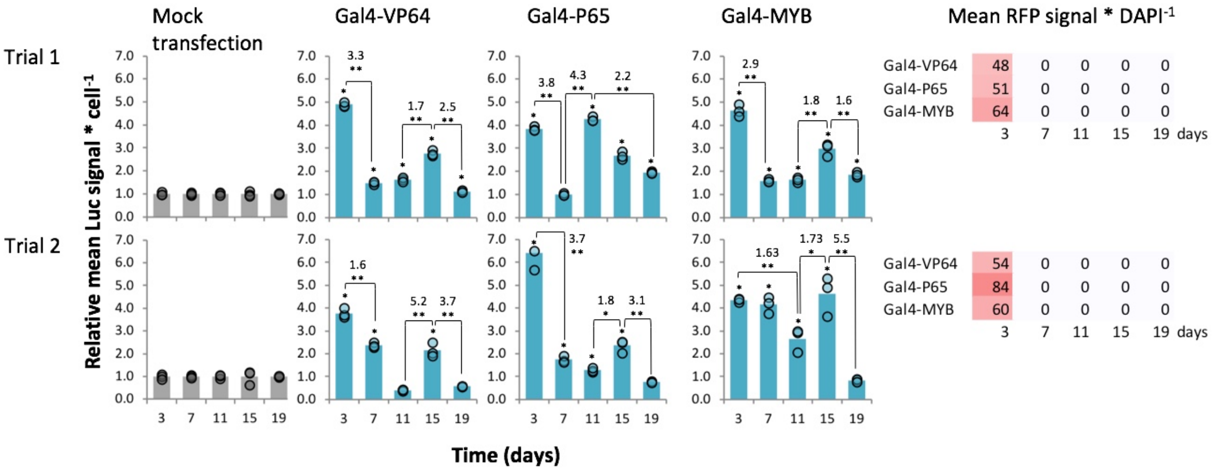

Figure S4. Additional trial of time course experiments with Gal4-AAP-expressing cells. Luciferase assays for Trial 2 were performed as described for Trial 1 from Figure 5B. As a simpler alternative to flow cytometry, a microplate reader was used to measure both RFP signal and DAPI signal from Hoescht-stained cells in 96-well plates *in situ*. The assay and data analysis were performed as we have described previously [S14]. Fold change values between time points for each Gal4-AAP experiment are shown within each bar graph. Different intra-group analyses (*t* tests) were done for Trial 1 versus Trial 2 in cases where large shifts in luciferase signal occurred at different days. Normalized RFP signal values are shown in each table (right). Asterisks represent *p*-values (* $p < 0.05$) for mean values greater than the mean for the mock-transfected negative control sample. * $p < 0.05$ and ** $p < 0.01$ are shown for intra-group comparisons (brackets).

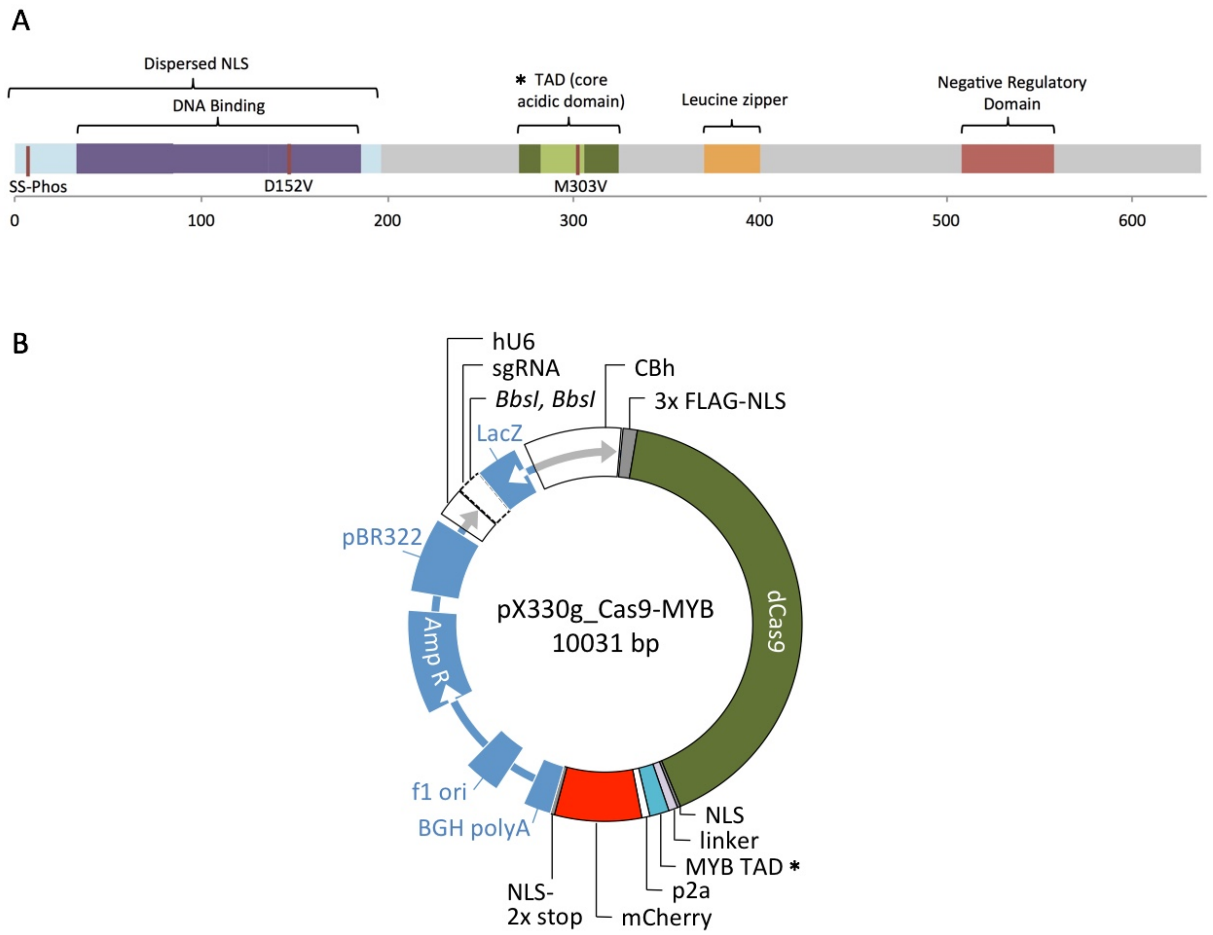

Figure S5. (**A**) Annotated motifs within the full-length MYB protein (AAA52032.1). From left to right: Dispersed nuclear localization signal (NLS) (M1-Q200; in light blue); Casein kinase II phosphorylation sites that reduce MYB DNA binding when phosphorylated and D152V mutation site that nullifies limited MYB pioneer function by disrupting DNA binding site recognition (S11, S12, D152; in red) [S15]; Repeat regions (R1-R3) that facilitate MYB binding to its recognition element (G34-L86, N87-L138, N139-M189; in purple) [S16, S17]; Transcription activation domain (P275-W327; in green) [S16]; Core acidic domain of TAD that facilitates interaction with CBP/p300 (D286-L309; in bright green) [S18]; M303V mutation that disrupts p300 recruitment and thus, activation by MYB (M303; in red) [S18, S19]; Leucine zipper domain that interacts with other cellular proteins (L383-L403; in orange) [S18]; Negative regulatory domain (V512-P566; in red), removal of which increases MYB-mediated expression increase [S20]. (B) Expression vector pX330g_dCas9-MYB was constructed from vector pX330A_dCas9 (a gift from Takashi Yamamoto, Addgene plasmid #63598) to co-express a dCas9-MYB fusion protein and mCherry from a CBh promoter. Single-stranded guide RNA sequences (Supplementary Table S4) were cloned into the BbsI sites and expressed from a hU6 promoter on the same vector.

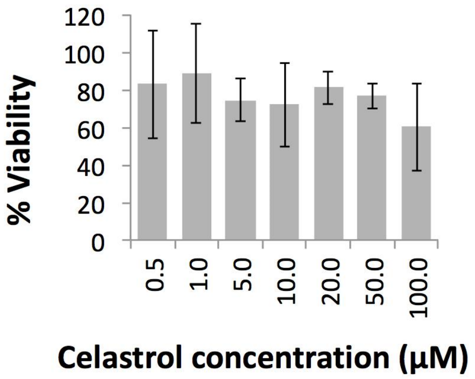

Figure S6. MTT cell viability assays. An MTT ((3-(4,5-Dimethylthiazol-2-yl)−2,5-diphenyltetrazolium bromide)) cell viability assay to determine the effects of celastrol on cell survival was performed as described previously by Godeshala et al. [S21]. In brief, Gal4-EED/luc cells were treated with celastrol diluted at different concentrations in Gibco DMEM high glucose. Cells were incubated with the drugs for six hours before being washed and cultured for 3 hours in drug free medium containing MTT reagent solution. Finally, cells were incubated with methanol:dimethyl sulfoxide (1:1) at room temperature for 30 minutes and mixed thoroughly before absorbance was read at 570 nm. The relative cell viability (%) was calculated from [ab]test/[ab]control × 100%. For each sample, the final absorbance/cell viability was reported as the average of values measured from three wells in parallel. Bars = standard error. We found no significant impact on cell viability after treatment with celastrol at any of the concentrations tested.

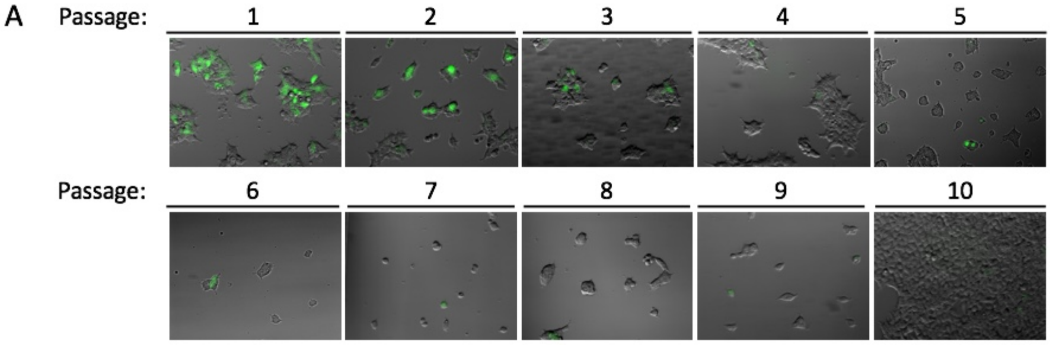

B

Passage	Mean GFP positive cell frequency (%)
1	55 ±20
2	47 ±14
3	20 ±6
4	11 ±4
5	11 ±8
6	15 ±8
7	13 ±9
8	3 ±2
9	4 ±4
10	< 2.5

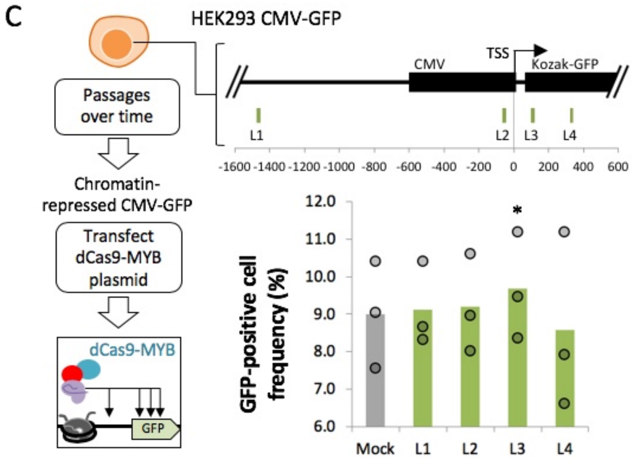

Figure S7. Targeting dCas9-MYB to a repressed CMV-GFP transgene in HEK 293 cells. (**A**) Fluorescence microscopy of HEK 293 CMV-GFP cells. Representative images (bright field and GFP channel overlay) at each passage are shown. The table shows mean frequencies of GFP-positive cells determined by manual counts of cells in the bright field channel and the GFP channel. Counts were carried out for three to four fields of view for each sample. (**B**) We targeted dCas9-MYB to four sites (L1-4) across a chromosomal *CMV-GFP* transgene in HEK 293 cells. Three days after transfection with dCas9-MYB/sgRNA vectors or mock transfection, we measured GFP fluorescence via flow cytometry. FlowJo software (version 10.6.1 for Mac OS X) was used to gate ~10,000 live cells (y = side scatter, x = forward scatter). These cells were then plotted as a histogram that showed a bimodal distribution of GFP signal (high mean GFP ~2.0E6, low mean GFP ~1.0E4). A bisector gate was used to determine the frequency of high-GFP cells in the live cell population. Circle = frequency of GFP-positive cells in one transfected sample; bars = means of three transfections. Asterisks (*) = $p < 0.05$ for experimental mean compared to the mock-transfected control mean.

SUPPLEMENTAL REFERENCES

1. Beerli RR, Segal DJ, Dreier B, Barbas CF 3rd. Toward controlling gene expression at will: specific regulation of the erbB-2/HER-2 promoter by using polydactyl zinc finger proteins constructed from modular building blocks. Proc Natl Acad Sci U S A. 1998;95:14628–33.

2. Liu PQ, Rebar EJ, Zhang L, Liu Q, Jamieson AC, Liang Y, et al. Regulation of an endogenous locus using a panel of designed zinc finger proteins targeted to accessible chromatin regions. Activation of vascular endothelial growth factor A. J Biol Chem. 2001;276:11323–34.

3. Zobel A, Kalkbrenner F, Vorbrueggen G, Moelling K. Transactivation of the human c-myc gene by c-Myb. Biochem Biophys Res Commun. 1992;186:715–22

4. Kawasaki H, Schiltz L, Chiu R, Itakura K, Taira K, Nakatani Y, et al. ATF-2 has intrinsic histone acetyltransferase activity which is modulated by phosphorylation. Nature. 2000;405:195–200.

5. Yang L, Wang H, Luo X, Mao P, Tian W, Shi Y, et al. Virion protein 16 induces demethylation of DNA integrated within chromatin in a novel mammalian cell model. Acta Biochim Biophys Sin . 2011;44:154–61.

6. Milne TA, Briggs SD, Brock HW, Martin ME, Gibbs D, Allis CD, et al. MLL targets SET domain methyltransferase activity to Hox gene promoters. Mol Cell. 2002;10:1107–17.

7. Nishioka K, Chuikov S, Sarma K, Erdjument-Bromage H, Allis CD, Tempst P, et al. Set9, a novel histone H3 methyltransferase that facilitates transcription by precluding histone tail modifications required for heterochromatin formation. Genes Dev. 2002;16:479–89.

8. Antonysamy S, Bonday Z, Campbell RM, Doyle B, Druzina Z, Gheyi T, et al. Crystal structure of the human PRMT5:MEP50 complex. Proc Natl Acad Sci U S A. 2012;109:17960–5.

9. Serandour AA, Avner S, Percevault F, Demay F, Bizot M, Lucchetti-Miganeh C, et al. Epigenetic switch involved in activation of pioneer factor FOXA1-dependent enhancers. Genome Res. 2011;21:555–65.

10. Clark KL, Halay ED, Lai E, Burley SK. Co-crystal structure of the HNF-3/fork head DNA-recognition motif resembles histone H5. Nature. 1993;364:412–20.

11. Wang W, Côté J, Xue Y, Zhou S, Khavari PA, Biggar SR, et al. Purification and biochemical heterogeneity of the mammalian SWI-SNF complex. EMBO J. 1996;15:5370–82.

12. Daer RM, Cutts JP, Brafman DA, Haynes KA. The Impact of Chromatin Dynamics on Cas9-Mediated Genome Editing in Human Cells. ACS Synth Biol. 2017;6:428–38.

13. Hansen KH, Bracken AP, Pasini D, Dietrich N, Gehani SS, Monrad A, et al. A model for transmission of the H3K27me3 epigenetic mark. Nat Cell Biol. 2008;10:1291–300.

14. Tekel SJ, Barrett C, Vargas D, Haynes KA. Design, Construction, and Validation of Histone-Binding Effectors in Vitro and in Cells. Biochemistry. 2018;57:4707–16.

15. Fuglerud BM, Lemma RB, Wanichawan P, Sundaram AYM, Eskeland R, Gabrielsen OS. A c-Myb mutant causes deregulated differentiation due to impaired histone binding and abrogated pioneer factor function. Nucleic Acids Res. 2017;45:7681–96.

16. George OL, Ness SA. Situational awareness: regulation of the myb transcription factor in differentiation, the cell cycle and oncogenesis. Cancers . 2014;6:2049–71.

17. Joaquin M, Watson RJ. Cell cycle regulation by the B-Myb transcription factor. Cell Mol Life Sci. 2003;60:2389–401.

18. Wang D-M, Lipsick JS. Mutational analysis of the transcriptional activation domains of v-Myb. Oncogene. 2002;21:1611–5.

19. Sandberg ML, Sutton SE, Pletcher MT, Wiltshire T, Tarantino LM, Hogenesch JB, et al. c-Myb and p300 regulate hematopoietic stem cell proliferation and differentiation. Dev Cell. 2005;8:153–66.

20. Gonda TJ, Favier D, Ferrao P, Macmillan EM, Simpson R, Tavner F. The c-Myb Negative Regulatory Domain. In: Current Topics in Microbiology and Immunology. 1996. p. 99–109.

21. Godeshala S, Nitiyanandan R, Thompson B, Goklany S, Nielsen DR, Rege K. Folate receptor-targeted aminoglycoside-derived polymers for transgene expression in cancer cells. Bioeng Transl Med. 2016;1:220–31.
